# β-Hydroxy-β-methylbutyrate (HMB) supplementation and functional outcomes in multi-trauma patients: a study protocol for a pilot randomised clinical trial (BOOST trial)

**DOI:** 10.1186/s40814-022-00990-9

**Published:** 2022-01-31

**Authors:** Kym Wittholz, Kate Fetterplace, Yasmine Ali Abdelhamid, Jeffrey J. Presneill, Lisa Beach, Benjamin Thomson, David Read, René Koopman, Adam M. Deane

**Affiliations:** 1 Department of Allied Health, The Royal Melbourne Hospital, 300 Grattan Street, Parkville, Melbourne, VIC 3050 Australia; 2 Department of Critical Care, Melbourne Medical School, The University of Melbourne, Melbourne, Australia; 3 Department of Intensive Care, The Royal Melbourne Hospital, Melbourne, Australia; 4 Department of Trauma and General Surgery, The Royal Melbourne Hospital, Melbourne, Australia; 5 Centre for Muscle Research, Department of Anatomy and Physiology, The University of Melbourne, Melbourne, Australia

**Keywords:** Critical illness, β-Hydroxy-β-methylbutyrate, Enteral nutrition, Trauma, Nutrition therapy, Muscle mass, Amino acids

## Abstract

**Background:**

There are no therapies proven to diminish the muscle wasting that occurs in patients after major trauma who are admitted to the intensive care unit (ICU). β-Hydroxy-β-methylbutyrate (HMB) is a nutrition intervention that may attenuate muscle loss and, thereby, improve recovery. The primary aim of this study is to determine the feasibility of a blinded randomised clinical trial of HMB supplementation to patients after major trauma who are admitted to the ICU. Secondary aims are to establish estimates for the impact of HMB when compared to placebo on muscle mass and nutrition-related patient outcomes.

**Methods:**

This prospective, single-centre, blinded, randomised, placebo-controlled, parallel-group, feasibility trial with allocation concealment will recruit 50 participants over 18 months. After informed consent, participants will be randomised [1:1] to receive either the intervention (three grams of HMB dissolved in either 150 ml of orange juice for those allowed oral intake or 150 ml of water for those being enterally fed) or placebo (150 ml of orange juice for those allowed oral intake or 150 ml of water for those being enterally fed). The intervention will be commenced in ICU, continued after ICU discharge and ceased at hospital discharge or day 28 post randomisation, whichever occurs first. The primary outcome is the feasibility of administering the intervention. Secondary outcomes include change in muscle thickness using ultrasound and other nutritional and patient-centred outcomes.

**Discussion:**

This study aims to determine the feasibility of administering HMB to critically ill multi-trauma patients throughout ICU admission until hospital discharge. Results will inform design of a larger randomised clinical trial.

**Trial registration:**

The protocol is registered with Australian New Zealand Clinical Trials Registry (ANZCTR) ANZCTR: 12620001305910. UTN: U1111-1259-5534.

## Background

Patients admitted to the intensive care unit (ICU) after a traumatic injury frequently suffer from a rapid reduction in muscle mass leading to substantial muscle weakness [1–4]. These changes in body composition and muscle strength are associated with prolonged hospital length of stay (LOS), increased mortality after ICU discharge, reductions in post-hospital discharge functional recovery and diminished quality of life (QOL) [1–5].

Various interventions have been evaluated in attempts to attenuate muscle loss in critically ill patients. These include nutritional interventions, such as increasing calorie [6, 7] and/or protein provision [6–9], providing amino acid supplementation [10, 11], enteral immunonutrition [12], physical interventions such as resistance exercise and/or early mobilisation [13, 14] and drugs, such as anabolic steroids [15]. However, the effects have been inconsistent [16–18]. This may be because the interventions chosen are truly of no benefit. Alternatively, the lack of effect may be due to the period of study. The majority of trials evaluating the impact of nutrition interventions in the critically ill are limited to the ICU admission [19]. Accordingly, the nutritional intervention is administered only for a short duration and during a period when enteral absorption of nutrients is most impaired. It is, therefore, biologically plausible that the provision of a nutrition intervention over an increased duration is more likely to attenuate muscle loss and improve functional outcomes when recovering from critical illness [20].

β-Hydroxy-β-methylbutyrate (HMB) is a metabolite of the essential branched-chain amino acid leucine [21]. HMB has been studied across a variety of adult populations from healthy athletes to conditions of muscle wasting such as cachexia, acquired immunodeficiency syndrome (AIDS), cancer, chronic obstructive pulmonary disease and critical illness [22, 23]. It appears to be a promising inexpensive agent that has been shown to affect muscle protein turnover by suppressing proteolysis [24] and stimulating protein synthesis [25]. A dose of 3 g of HMB per day is associated with preservation of lean body mass in healthy older adults [26] and it appears to have no adverse health outcomes [27, 28]. In a blinded, randomised controlled trial of 19 healthy older adults, HMB administration attenuated the decline in lean body mass over 10 days of bed rest (HMB group lost an average of − 0.17 ± 0.19 kg total lean mass (*p* = 0.42, paired *t* test) versus control (− 2.05 ± 0.66 kg, *p* = 0.02, paired *t* test); *p* = 0.02, ANOVA) [26]. Given those with critical illness experience prolonged bed rest, HMB supplementation is a candidate nutritional intervention to attenuate muscle loss and weakness associated with critical illness.

Bear and colleagues conducted a systematic review and meta-analysis of randomised control trials that used HMB as a single agent or as part of a supplement [23]. When including a variety of cohorts, they reported that HMB administration improved muscle mass and strength [23], albeit all studies had high risk of bias and effect size was small.

Hitherto, only three randomised clinical trials have evaluated the effect of HMB supplementation in critically ill populations [29–31]. All three trials limited the period of intervention to the ICU admission. In a single centre trial of 100 severely ill trauma patients, Kuhls and colleagues reported that 3 g/day of HMB, when compared to placebo, markedly attenuated negative nitrogen balance [29]. Hsieh and colleagues examined the impact of HMB on inflammatory markers in critically ill chronic obstructive pulmonary disease (COPD) patients. They reported white blood cell count, C-reactive protein and creatinine to be significantly lower, whilst cholesterol and total protein were significantly higher after HMB supplementation [30]. However, neither study investigated the effect of HMB on muscle mass or physical function. Nakamura and colleagues [2] evaluated the impact of HMB in conjunction with arginine and glutamine when compared to control (no HMB supplementation) on change in muscle volume using computed tomography during the ICU admission in 88 severely ill medical or surgical patients [31]. Both groups were observed to lose muscle volume over the acute 10-day study period with point estimate favouring a greater volume of femoral muscle retained in those receiving HMB [31].

To summarise, it is not known whether nutritional HMB supplementation will attenuate muscle loss and improve outcomes in patients recovering from major trauma. It is also unclear if it is feasible to conduct a trial of a blinded nutrition intervention in the ICU and continue after ICU discharge, whilst collecting adequate outcome data.

### Study objectives

The objective is to determine the feasibility of undertaking a blinded randomised clinical trial of HMB supplementation to critically ill multi-trauma patients until day 28 post randomisation or hospital discharge. Feasibility will be established by evaluating:

- An ability to blind the intervention.
- The recruitment and retention rates according to the study methods.
- The ability to perform the outcomes measures within this patient cohort weekly until day 28 post randomisation or hospital discharge whichever occurs first and again at day 90 post enrolment.
- The extent to which mortality may be a competing risk in this patient cohort for nutritional and functional outcome assessments.

Secondary objectives are:

- To return initial estimates of effect size and variance associated with HMB treatment on muscle mass.
- To return initial estimates of effect size and variance associated with HMB treatment on other nutrition-related patient outcomes including changes in weight, nutritional status, nutrition intake, appetite, muscle strength, physical function and quality of life in a multi-trauma population compared to standard care.

## Methods/design

**Fig. 1:**
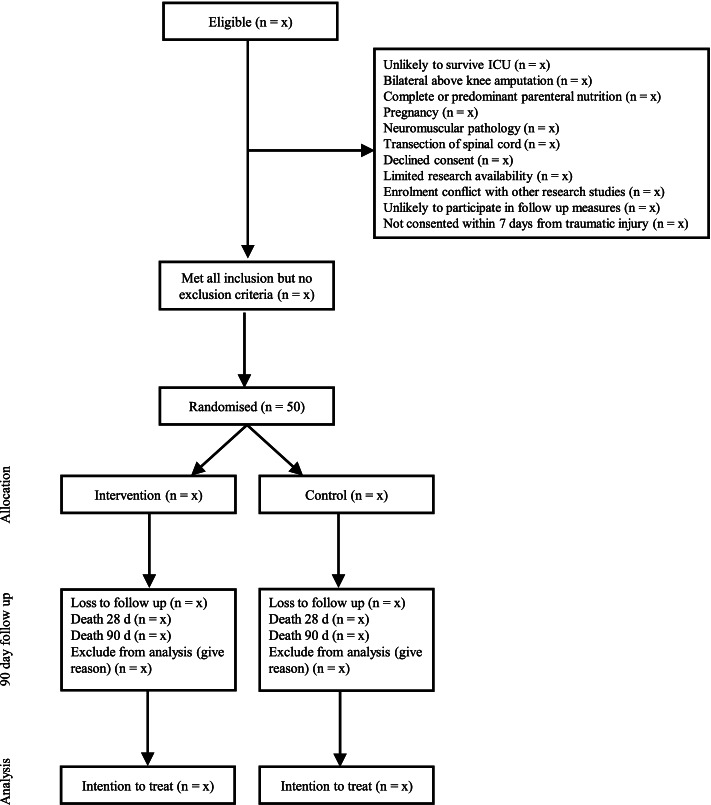
Modified consort

This will be a prospective, single-centre, placebo-controlled, two parallel-group, randomised feasibility trial with allocation concealment and blinded assessors. The design is in accordance with the Standard Protocol Items: Recommendations for Interventional Trials (SPIRIT 2013) [32] and the Consolidated Standards for Reporting of Trials CONSORT guidelines [33] (Fig. 1, modified consort). The study will be undertaken at the Royal Melbourne Hospital, which is a university-affiliated, trauma referral centre in Victoria treating up to 1000 patients per year with major trauma.

### Study participants

Fifty participants who are admitted to the ICU due to traumatic injury will be recruited over an 18-month period (September 2020–February 2022). If participant recruitment is significantly less than this, the study can be extended for an additional 6–12 months. Patients will be screened following admission to ICU and identified as eligible according to the criteria presented in Table 1.

**Table 1:** Inclusion and exclusion criteria

**Inclusion:** Adults, ≥ 18 years of age Completed two full calendar days in ICU The predominant reason for ICU admission was a traumatic injury Allowed enteral/ oral nutrition at the time of randomisation	
**Exclusion:** Death during ICU admission deemed to be inevitable Bilateral above knee amputation Patients assessed as requiring completely or predominantly parenteral nutrition Pregnancy Primary neuromuscular pathology present or strongly suspected this admission episode Presumed transection of the spinal cord at any level Medical decision treatment maker, participant or medical practitioner declined consent Limited research availability over enrolment timeframe Enrolment conflict with other research studies Unlikely to be able to participate in long term follow up measures Unable to obtain consent within 7 days from initial traumatic injury	

For study recruitment, consent will be sought from patients wherever they can give consent and it is practicable to approach them. Where it is not practicable to approach a person highly dependent on medical care, or the person is not capable of making such a decision, informed consent will be obtained from the person responsible as per local laws [34]. Consent to continue in the trial will be obtained from the participant if they recover adequately and they are deemed competent. The protocol and consent process has been approved by the Royal Melbourne Hospital Human Research Ethics Committee (2019.358). The protocol is registered with Australian New Zealand Clinical Trials Registry (ANZCTR 12620001305910).

### Randomisation, allocation concealment and blinding

Following enrolment, participants will be randomised by a designated research scientist who has no role in the trial to either the intervention or control group using a [1:1] ratio allocation within permuted blocks. The randomisation sequence was created using the R Package randomizeR [35] and is concealed from the staff involved in enrolment, consent and all data collection. The randomisation sequence is protected by an electronic password known only to the designated research scientist. The intervention and control solution will be prepared by the designated research scientists who are not involved in the collection of outcome measures or other study procedures. All other members of the participants treating team, including doctors, nursing staff, dietitians and physiotherapists, are blinded to the treatment group.

### Baseline data collection

Following enrolment, baseline data will be collected. This includes demographic data (age and sex), pre-morbid place of residence and employment status, Katz Activities of Daily Living (ADL) index [36] (prior to the ICU admission), illness severity (Acute Physiology, Age, Chronic Health Evaluation II (APACHE II) [37], Australian and New Zealand Risk of Death score (ANZROD) [38] and Injury Severity Scale (ISS) [39], baseline anthropometric data (weight, height, body mass index (BMI)) and nutrition status (assessed using the Subjective Global Assessment (SGA)) [40, 41].

### Standard nutrition practice

At this facility, nutrition provision is commenced in the ICU according to a protocol (Appendix 17). Nutrison Protein Plus® 1.25 kcal, Nutricia, Wuxi, China), providing 63 g protein and 1250 kcal per litre, is the standard nutrition formula and will be commenced at 25 kcal/kg of ideal body weight (IBW) [42]. IBW is defined as body mass index (BMI) between 18.5 and 25 kg/m^2^ for 18–65 years and 22–27 kg/m^2^ for > 65 years. For underweight participants, actual weight will be used. For obese patients with a BMI > 32 kg/m^2^, IBW + 25% (actual weight – IBW) will be used [43]. For patients who are likely to remain intubated for > 7 days and do not meet contraindications [44], a dietitian will measure energy expenditure (MEE) using indirect calorimetry using E-sCOVX (GE, Helsinki, Finland) [45]. Weight-based equations are used to determine estimated protein requirements and are set at 1.2–2.0 g/kg/day [42]. A dietitian will regularly assess nutrition requirements and adjust protein and energy targets on an individual basis as clinically indicated as part of standard nutrition care at this facility.

### Trial intervention and control

The trial intervention is β-hydroxy-β-methylbutyrate (HMB). HMB has been purchased from Myprotein (Warrington, UK). Three grams of HMB is dissolved in 150 ml of fluid. To ensure blinding of participants is maintained, orange juice is the fluid for those allowed oral intake and water for those being enterally fed. Orange juice completely masks the taste and smell of the HMB supplement. HMB powder dissolves completely in water and does not impact the viscosity of the solution. The control is 150 ml of orange juice for those allowed oral intake, or 150 ml of water for those being enterally fed. If a patient is restricted to ingesting only thickened fluids and unable to receive the intervention/control via an enteral feeding tube, Flavour Creations™ orange juice thickened to the appropriate viscosity will be used to blind the intervention/control.

Patients will receive the intervention or control from day 1 post enrolment until hospital discharge or study day 28, whichever occurs first. If a patient is readmitted to hospital within 28 days from randomisation, study procedures and administration of the intervention or control will resume. The intervention or control will be documented in the electronic medication record by the treating team and administered by the nurse caring for the patient. The number of doses prescribed and consumed will be recorded for the duration of the study. The nurse caring for the patient will document patient compliance with the intervention or control and any tolerance issues voiced by the patient believed to be associated with the consumption of trial supplement. The reason for any missed doses will be recorded.

The investigator/s, patient, nurse and member of the treating team will be surveyed on day 1 post enrolment and then day 28 or hospital discharge, whichever occurs first, as to their blinding to the intervention/control as part of the feasibility criteria. All will be invited to take their best guess of whether the participant is receiving the intervention or placebo.

### Outcome measures

Primary and secondary outcome measures will be captured by trained study investigator/s. Summary study schedule and outcome measures are detailed in Table 2. Any outcome data for weight, nutrition status, appetite, muscle mass, muscle strength or physical function, collected within ± 48 h of the specified time points from day 0 to day 28 will be used for this feasibility study. For practical reasons, it may not always be possible for patients to return to hospital on the exact 90-day follow-up time point. Therefore, any data collected within a 2-week window of the “90 day” follow-up measure will be used, and date of measure recorded.

**Table 2:** Study schedule and outcome measure

	Assessment/Procedure	Screening and enrolment (day 0)	Day 1, then weekly until day 28 or hospital D/C	Daily from day 1–day 28 or hospital D/C	Day 1	Day 0 until ICU D/C and then 7 days post ICU D/C	ICU D/C	Day 28 or hospital D/C	Day 90 post enrolment
**Study procedures**	Informed consent	**X**							
	Demographic data, APACHE II, ANZROD and ISS, baseline creatinine and urea	**X**							
	Anthropometric data (weight, height and BMI)	**X**							
	Body weight		**X**						**X**
	Nutrition status (SGA)	**X**						**X**	**X**
	Muscle mass		**X**						**X**
	Handgrip strength		**X**						**X**
	Physical function (IMS and mILOA)						**X**	**X**	
	Physical function (PFIT-s)						**X**		
	Patient-reported appetite		**X**						**X**
	Nutrition intake					**X**			
	Administration of intervention/control			**X**					
	Blinding survey				**X**			**X**	
	ICU LOS, days of mechanical ventilation, hospital LOS, discharge destination, use of renal replacement therapy, highest creatinine and urea							**X**	
	Quality of life survey								**X**
	Place of residence and employment status	**X**							**X**
	Mortality							**X**	**X**

*ANZROD* Australian and New Zealand Risk of Death score, *APACHE II* Acute Physiology, Age, Chronic Health Evaluation II, *BMI* body mass index, *IMS* ICU Mobility Scale, *ISS* Injury Severity Scale, *LOS* length of stay, *mILOA* modified Iowa Level of Activity, *PFIT*-*s* Physical Function ICU Test-Scored, *SGA* Subjective Global Assessment

#### Primary outcomes

The feasibility of administering the intervention or control will be quantified as:

- Successful blinding of the intervention determined through patient and clinician surveys
- Recruitment and retention rates analysed for patients who meet all inclusion and none of the exclusion criteria
- The amount of HMB supplementation actually consumed compared to the amount intended with full protocol compliance

The following feasibility indicators will be reported:

- Percentage of surveyed clinicians are able to correctly identify the intervention
- Percentage of patients who meet all of the inclusion and none of the exclusion criteria are recruited and % of enrolled patients retained until hospital discharge; and
- Percentage of the prescribed HMB supplementation doses consumed

#### Secondary outcomes

Ultrasound will be used to determine muscle mass on day 1 of enrolment and then weekly until day 28 or hospital discharge whichever occurs first and again at day 90 post enrolment where possible. An additional muscle mass measurement will be completed if there is no measurement scheduled within 48 h of ICU and hospital discharge. Ultrasound of leg muscularity in critically ill populations is an emerging non-invasive bedside technique that has been shown to predict muscle mass at the 3rd lumbar vertebra when quantified with computed tomography (CT) [46–48]. Measurement of muscle thickness with ultrasound is therefore a validated technique for assessment of skeletal muscle mass with good inter- as well as intra-rater reliability [46–48]. The ultrasound technique used in this protocol has been reported to be a safe and reliable measure of muscle mass [49–51]. A Philips Lumify portable ultrasound device available in the RMH ICU will be used to obtain muscle mass images. The method to obtain the images will be carried out as previously described [46, 47]. The quadriceps muscle layer thickness (QMLT) will be measured on the right side at two points; the midpoint between the anterior superior iliac spine (ASIS) and the upper pole of the patella and at the point 2/3 between the ASIS and the top of the patella. The ultrasound transducer will be held perpendicular to the skin and depth standardised at 6 cm or adjusted to visualise the femur. Three frozen images will be recorded using minimal pressure and three frozen images will be recorded using maximal pressure at each site. On screen callipers will be used to record muscle thickness and the average distance will be recorded. The upper arm muscle thickness (UAMT) will be measured on the right side at the midway point between the tip of the acromion and olecranon process [52]. Three linear still images will be taken at each landmark and recorded. Ultrasound measures will be completed on the left side if the right side is unavailable. Muscle mass measurements will be reported as change adjusted for baseline measurements [8].

Interrater reliability testing of ultrasound measures will be conducted to ensure consistency of this outcome measure. A second trained assessor will repeat the baseline land marking and image acquisition in a total of 5 patients, selected at random. In addition, interrater reliability for the quantification of muscle thickness will occur using a trained external assessor who will complete a second analysis of baseline images in 5 patients, selected at random. The muscle thicknesses for each set of images in the two subgroups will be compared [49].

Handgrip dynamometry will be used to assess muscle strength [53–55]. Handgrip dynamometry will be completed on day of enrolment and then weekly until day 28 or hospital discharge whichever occurs first and again at day 90 post enrolment. Handgrip dynamometry (Commander Echo™ Wireless Grip Dynamometer, USA or Jamar Digital Plus™, USA) will be measured in both limbs and repeated three times. Participants will be sitting in a chair or sitting at least at 45° in bed, with the patients elbow at 90° supported by a pillow or the arm of the chair. The highest measure will be recorded. An additional muscle strength measurement will be completed if there is no measurement scheduled within 48 h of ICU and hospital discharge. The highest score will be recorded.

Weight will be measured using bed scales, chair scales, hoist scales or standing scales as appropriate. Weight will be measured on day of enrolment and then weekly until day 28 or hospital discharge whichever occurs first and again at day 90 post enrolment. Weight will be reported as change adjusted for baseline.

The Subjective Global Assessment (SGA) will be used to determine the proportion of patients diagnosed with malnutrition within 48 h of hospital discharge and 90 days post enrolment adjusted for baseline nutritional status [40, 41, 53]. Those patients who have an SGA score of B (mild-moderate) or C (severe) will be classified as malnourished.

Nutrition intake from all sources including dextrose, propofol, parenteral nutrition, enteral nutrition documented in the fluid balance chart and oral intake documented in food record charts will be recorded from day of enrolment until ICU discharge and then for a total of 7 days post ICU discharge (where day of ICU discharge is defined as day 1). Energy and protein intake from all sources will be calculated by the dietitian and compared to estimated energy and protein requirements to determine nutrition adequacy over this time.

Patient-reported appetite will be assessed using visual analogue scales (VAS) [56, 57]. This will be assessed on day 28 or hospital discharge whichever occurs first and again at day 90 post enrolment.

Physical function will be assessed using the Physical Function ICU Test-Scored (PFIT-s) [58] at ICU discharge. Medical records will be retrospectively reviewed to obtain any other PFIT-s score completed over the ICU admission as part of routine care by a physiotherapist. The ICU mobility scale (IMS) [59] and modified Iowa Level of Activity (mILOA) [60] scale will be used to investigate the highest level of activity at ICU discharge and hospital discharge. The EQ-5D-5L questionnaire will be used to assess patient-rated quality of life at day 90 post enrolment [7, 61].

Computed tomography (CT) of the skeletal mass cross-sectional area will be used as an additional measure of muscle mass for a subgroup of patients who have one or more abdominal CT scans ordered as part of their medical treatment at any time over their hospital admission [46, 47]. A trained radiologist will determine if all components of the skeletal mass can be assessed at the level of L3 of an abdominal CT scan [47]. If the scan is deemed suitable, the scan will be downloaded for analysis using the Automated Muscle and Adipose Tissue Composition Analysis (AutoMATiCA) program which provides an automated analysis for skeletal muscle cross-sectional area (CSA) [46, 62]. The patients will be categorised as having low muscularity if the skeletal muscle CSA is < 110 cm^2^ for women and < 170 cm^2^ for men [63]. Changes in skeletal muscle CSA will be determined by comparing the difference between scans and the length of time this occurred over will be determined and recorded. The date of the CT scan and corresponding day of study will be recorded.

Data regarding the duration of admission, days of mechanical ventilation, any use of renal replacement therapy and highest markers of kidney function (urea and creatinine) from day of enrolment until day 28 post enrolment, hospital length of stay, in-hospital mortality and discharge destination will be collected.

All data collected by the investigator/s will be entered into an electronic database (REDCap). Data will be collected from day of enrolment until day 90.

### Management of adverse events

It is not anticipated that any adverse events will occur in relation to the study protocol. If any adverse events do present, the nature, severity, causality and course of the adverse event will be recorded. All adverse events will be recorded from the time of consent; any event will be discussed with the attending Intensivist or Trauma consultant. Any serious adverse events related to the study will be reported by the investigator to the Melbourne Health Human Research and Ethics Committee within 24 h of site personnel becoming aware of it.

### Withdrawals

A participant (or his/her surrogate decision maker) may choose to withdraw at any stage by choice or if they experience an adverse event. If at any time the attending consultant feels that the intervention or control oral supplement is inappropriate for the patient, the patient can be withdrawn from the study. Any patient who is withdrawn from the study will not be replaced.

### Sample size

To our knowledge, this will be the first study to report feasibility of HMB administration to critically ill trauma patients. Therefore, data will be used to generate sample size estimates for later confirmatory studies [64, 65]. For this trial, a suitable number of participants has been estimated based on admission data at the host institution. A recent observational study was able to recruit 28 patients over a 6-week period using a similar eligibility criteria [4]. Accounting for attrition associated with obtaining written informed consent, it is estimated to be feasible to recruit 50 participants over a 18-month period [65]. If participant recruitment is significantly less than this, the study can be extended for an additional 6–12 months.

### Analysis plan

Baseline variables including demographics, severity of illness, ICU and hospital length of stay, mortality and nutritional markers will be reported according to the two randomised groups. Differences between the two treatment groups will be reported for the multiple parameters of interest, including change in weight, muscle mass and muscle strength from baseline to day 28 or hospital discharge. As well, the intraclass correlation coefficient (ICC) and coefficient of variation (CV) will be used to summarise interrater reliability of the ultrasound measurements.

Selected differences among these parameters of interest will be compared between randomised groups using the two-sample unpaired *t* test for approximately normal data, the Wilcoxon-rank sum test for substantially skew data and the Fisher’s exact test for categorical variables within contingency tables as appropriate. As well, exploratory multivariable models may be generated, incorporating adjustment for initial baseline values and other selected potentially relevant co-variables using linear regression. Results will be reported with 95% confidence intervals to aid in the interpretation of results in the context of pilot data.

Given the illness severity of the trauma patients under study, deaths during the study are likely, making the outcomes often only practically ascertainable in survivors. In the presence of sufficient data, exploratory time-to-event analyses in competing-risks regression models may be applied to return the subhazard functions of failure events of primary interest whilst accounting for the competing outcome of death using the method of Fine and Gray [66]. Multiplicity of testing will be acknowledged but multiple statistical tests will be otherwise unadjusted, consistent with the exploratory nature of this pilot study. Data analyses will be carried out using recent versions of established statistical software packages, which at the time of writing include the Statistical Package for the Social Sciences (IBM® SPSS® Statistics) and Stata (Stata Statistical Software. College Station, TX: StataCorp LP; 2019).

## Discussion

There is no accepted intervention to prevent or attenuate muscle wasting in critically ill trauma patients [16–18]. Provision of a nutrition intervention over an increased duration may be more likely to attenuate muscle loss and improve patient-centred outcomes when recovering from critical illness [20]. HMB is an inexpensive nutritional intervention that has been shown to positively affect muscle mass and strength in similar clinical populations and may, therefore, be effective to accelerate recovery in patients after major trauma [23].

The aim of this study is to determine the feasibility of undertaking a large blinded randomised control trial of HMB supplementation to critically ill multi-trauma patients until day 28 post randomisation or hospital discharge. Results will inform sensitivity analysis of the potential for intervention effectiveness, and quantification of feasibility in the form of completeness of data, recruitment and retention rates.

Strengths of the study include the randomised design and the blinding of clinicians, patients and assessors to the intervention. Limitations of the study include the single-centre design and possible differences in the duration of intervention or control administration.

## Figures and Tables

**Fig. 2:**
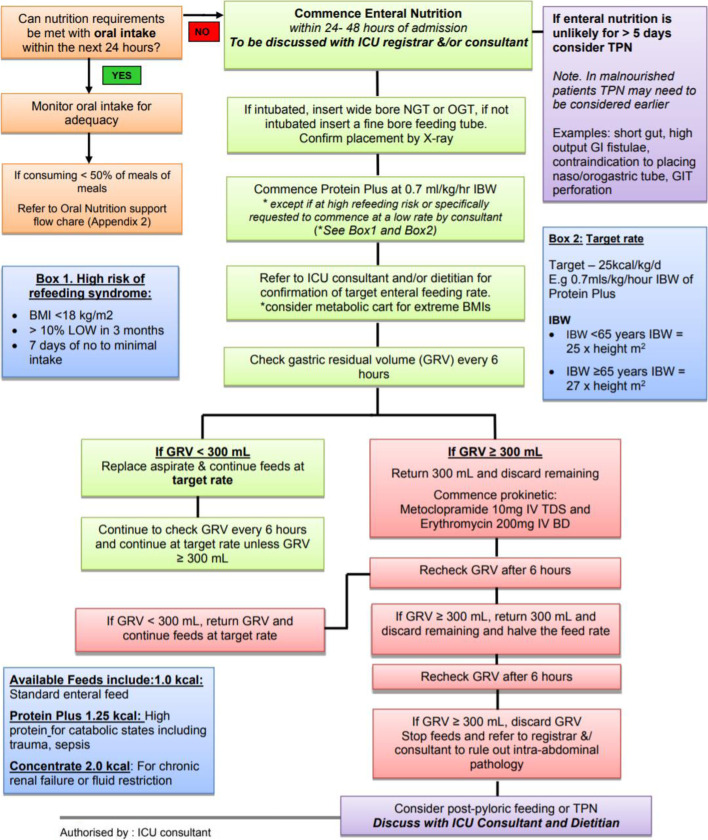
ICU nutrition and enteral feeding flowchart

## Data Availability

The datasets used and/or analysed during the current study are available from the corresponding author on reasonable request.

## Appendix

Figure 2
